# Drug resistant HIV: Behaviors and characteristics among Los Angeles men who have sex with men with new HIV diagnosis

**DOI:** 10.1371/journal.pone.0173892

**Published:** 2017-03-23

**Authors:** Pamina M. Gorbach, Marjan Javanbakht, Lorelei Bornfleth, Robert K. Bolan, Martha Lewis Blum

**Affiliations:** 1 Department of Epidemiology, Fielding School of Public Health, University of California, Los Angeles, CA, United States of America; 2 Division of Infectious Diseases, Department of Medicine, David Geffen School of Medicine, University of California, Los Angeles, CA, United States of America; 3 The Los Angeles Gay, Lesbian and Transgender Health Center, Los Angeles, CA, United States of America; 4 Department of Medicine, Community Hospital of the Monterey Peninsula, Monterey County, Department of Public Health, Monterey, CA, United States of America; Fudan University, CHINA

## Abstract

Epidemiology of drug resistant HIV has focused on trends and less attention has been given to identification of factors, especially behaviors including substance use, in acquisition of drug-resistant HIV. From 2009 to 2012 The Metromates Study enrolled and followed for one year men who have sex with men (MSM) seeking testing for HIV in a community clinic in Los Angeles assessing those testing positive for acute and recent HIV infection. Behavioral data were collected via Computer-Assisted Self-Interview from 125 classified as newly HIV infected and 91 as chronically infected (newly HIV-diagnosed); specimens were available and viable for resistance testing for 154 of the 216 HIV positives with new diagnoses. In this community clinic we found prevalence of resistance among MSM with new HIV-diagnosis was 19.5% (n = 30/154) with no difference by recency of HIV infection. Sexual partnership characteristics were associated with resistance; those who reported transgendered sex partners had a higher prevalence of resistance as compared to those who did not report transgendered sex partners (40% vs. 17%; p value = 0.04), while those who reported having a main partner had a lower prevalence of drug resistance (12% vs. 24%; p value = 0.07). In multivariable analyses adjusting for HIV recency and antiviral use, reporting a main partner decreased odds [adjusted odds ratio (AOR) 0.34; 95% confidence interval (CI) 0.13–0.87], reporting a transgendered partnered increased odds (AOR = 3.37; 95% CI 0.95–12.43); and being African American increased odds of drug resistance (AOR = 5.63, 95%CI 1.41–22.38). This suggests African American MSM and TG individuals in Los Angeles represent pockets of exceptional risk that will require special approaches to prevention and care to enhance their own health and reduce their likelihood to support transmission of drug resistance in the US.

## Introduction

Among those with a new diagnosis of HIV infection, some are infected by a strain of HIV-1 resistant to one or more drugs, known as primary or transmitted drug resistance (TDR) [1]. The efficiency of such transmission between individuals is relatively low[2] but evidence is growing that such resistance can stem from untreated individuals and strains can persist in populations for many years[3, 4]. In a meta-analysis the median overall TDR prevalence globally ranged from 2.8% to 11.5%, and non-nucleoside reverse transcriptase inhibitor (NNRTI) associated TDR increased in North America[5]. Levels of TDR in the US and Europe among men who have sex with men (MSM) appear similar although pockets of higher prevalence ranging from 12–24% have been documented[6, 7] [8]. There have been few studies of behavioral factors associated with resistance [9, 10]. There are conflicting findings about substance use. Methamphetamine use was associated with acquisition of multi-drug resistant virus in New York in 2005[11] and we confirmed this in 2008 along with other substance use in Southern California[9]. However, other studies have not shown association of substance use and resistance even among injection drug users[12]. To contribute to understanding the role of substance use and other risk factors in acquisition of drug-resistant HIV we conducted a study of MSM with new HIV diagnoses in Los Angeles in a community clinic setting.

## Materials and methods

Between 2009–2012 321 MSM enrolled in the NIDA funded Metromates Cohort Study conducted at The Los Angeles LGBT Center, a community-based organization providing sexual health services, social services and HIV care in Los Angeles. Men at least 18 years of age reporting sex with a man in the past 12 months who sought HIV testing at the LGBT Center were offered an opportunity to undergo a consent process including a written consent with a full description of the study with choices about if and how to participate in specimen donation, and then were provided a copy of their written consent form (the original was saved with study materials). Those who tested HIV-positive provided a specimen for testing of recency of infection using the Vironostika detuned assay (BioSystems Laboratory in San Francisco) a validated assay for use of detection of early HIV infection[13, 14]; negative antibody results were tested for acute infection using RNA nucleic acid amplification testing (NAAT). Those testing positive (n = 216) were all verified as newly diagnosed, with a subset classified as recently infected (125) using one of the following four criteria: (1) documentation of a negative HIV test <12 months prior to enrollment; (2) a NAAT positive test result on a specimen negative by HIV antibody testing; (3) result indicative of recent infection from a detuned serologic assay; and (4) documentation of recent infection by a referring physician. The remaining 91 who had a positive rapid HIV test yet showed no evidence of recent infection using the assays described above were classified as newly HIV-diagnosed (but not newly infected) and 105 were HIV-negative. A subset of the HIV-positive men (173/216) consented to providing plasma samples for storage for future analysis and 154 of these were viable for resistance testing (n = 17 of the newly diagnosed did not have sufficient viral load for genotyping and two were missing baseline data). Analysis was restricted to 154 men with resistance testing data. No differences noted between those excluded and the analyzed sample except for more testing done in those without a main partner compared to those with (76.2% vs. 64.6; p value = 0.05).

### Behavioral data

A Computer-Assisted Self-Interview (CASI) assessed sexual partner type (main partner, casual partner, female partner, and transgender partner); types of sexual activity (group sex and transactional sex); substance use (marijuana, cocaine, inhalants, opioids, methamphetamine, and injection drug use). Also assessed was use of ARV drugs for treatment, pre-exposure prophylaxis (PrEP) or post-exposure prophylaxis (PEP), substance use, and sexual behavior. “Treatment status” was determined from reports of date starting ARVs as some men started ARVs before enrollment due to a lag between communication of an HIV positive test result and study enrollment. Initiation of care was not an exclusion criteria for this analysis. The questions and scales used in this study were developed from two previous studies of men with acute HIV infection or previous diagnosis; with careful attention to included comparable questions/measures to the previous cohort that identified behavioral factors associated with resistant HIV [15–17]. The Partnership Assessment Scale and definitions of partnership types were developed using sociometric analyses in previous studies and research on partnerships in general as well as qualitative research [18].

### Resistance testing

HIV genotyping was performed using stored plasma samples collected at study enrollment. The testing of those assessed as acutely infected (n = 104) was conducted at the HIV Genotyping Laboratory at Johns Hopkins University (JHU) and the other new diagnoses at University of California Los Angeles (UCLA) School of Medicine (n = 69). Testing at JHU was conducted using the ViroSeq HIV Genotyping System v2.8 (Celera Diagnostics, Alameda, CA). This system provides HIV sequences for the region encoding HIV protease (amino acids 1–99) and HIV reverse transcriptase (amino acids 1–335). Testing at UCLA was done using the UltraSens Viral Isolation kit (Qiagen) according to the manufacturer’s protocol. The Reverse Transcriptase (RT) and Protease (PRO) genes were amplified and sequenced according to the previously published protocol of Snoeck et al. using SuperScript III RT (Invitrogen) for RT and KOD high fidelity polymerase (Novagen) for PCR. PCR products were directly sequenced using the BigDye v3.1 Kit (Applied Biosystems) according to the manufacturer’s protocol using the primers described in Snoeck et al[19]. Sequences were edited and assembled using BioEdit (Ibis Biosciences, Carlsbad, CA). ARV drug resistance was determined by consulting the Stanford University HIV Drug Resistance Database (http://hivdb.stanford.edu/). Samples were classified as “resistant” if results indicated reduced sensitivity to at least one class of ARVs.

### Statistical methods

Differences in ARV drug resistance to at least one class of HIV drugs by demographics and behaviors were evaluated using t-tests, Wilcoxon rank sum, chi-square methods, Fisher’s exact tests and multivariable logistic regression analysis. Behaviors included in the analyses were selected based on findings from the research team’s previous work with this population and this topic[9, 17] as well as the existing literature. All analyses were conducted using SAS software, version 9.2 (SAS Institute Inc., Cary, NC). Study procedures were reviewed and approved by the UCLA IRB and the LGBT Center’s Research Committee.

## Results

Among the 154 recently HIV-diagnosed MSM with testable plasma the mean age was 30 years, nearly half identified as Hispanic (48%), followed by white (29%) and African American (17%); 58% were identified as having an acute/recent HIV-infection. The median number of sexual partners in the past 12-months was eight [interquartile range (IQR) 4–20], with a median of two anonymous partners and one one-time partner. More than half the participants (n = 79; 52%) reported substance use in the past 3 months, including marijuana (41%), methamphetamine (20%), cocaine (15%), and opioids (3%). Eleven (7%) reported sex with both men and women; and 30 (19.5%) reported a transgender partner in the last 12 months; 30 (19.5%) reported transactional sex in the past 3 months. Between screening and enrollment 30 men (19%) had initiated ARV therapy. Among these HIV positive men more methamphetamine use was reported among newly infected than chronically infected (33% vs. 20%; p value = 0.07).

Drug resistance to at least one class of ARVs was identified in 30 (19.5%) of the 154 participants. Most of the resistance was to NNRTIs: 25/30 cases of resistance. The prevalence of ARV resistance did not vary significantly by HIV diagnosis group (Table 1) although the higher prevalence among those newly diagnosed versus newly infected (24.6% versus 16.1%) may have been significant in a larger sample size. There was no significant difference in prevalence of resistance between between those reporting ARV use and reporting no ARV use (22.2% versus 19.4%, respectively; p value = 0.76). Futhermore, when we excluded the 20 reporting ARV use resistance prevalence did not change, remaining 19.4%. Sexual partnership characteristics were associated with resistance; those who reported transgendered sex partners had a higher prevalence of resistance as compared to those who did not report transgendered sex partners 40% vs. 17%; p value = 0.04), while those who reported having a main partner had a lower prevalence of drug resistance (12% vs. 24%; p value = 0.07). No other behavioral factors were associated with resistance including number of sexual encounters, unprotected anal intercourse (UAI), and having a known HIV-positive partner. There were more methamphetamine users with resistance but the difference was not statistically significant (25.8% vs. 18.2%; p value = 0.34). In multivariable analyses adjusting for HIV group (recent or chronic infection) were conducted with and without those reporting ARV use; and the ARV use was adjusted for in the models in which those individuals were included (Table 1).

**Table 1:** Prevalence and factors associated with antiretroviral drug resistance among men newly infected and/or newly diagnosed with HIV, Los Angeles, 2009–2012 (n = 154).

	n / N	%	P value	OR	(95% CI)	AOR	(95% CI)	AOR (excluding those on ARVs n = 22)	(95% CI)
Age, years^			0.78	1.01	(0.96–1.06)				
Resistance	30.8 (8.8)					—	—	—	—
No resistance	30.4 (7.5)					—	—	—	—
Race/ethnicity			0.09						
African American	9 / 23	39.1		4.07	(1.22–13.53)	5.63	(1.41–22.38)	4.40	(1.05–18.40)
Hispanic	14 / 73	19.2		1.50	(0.53–4.25)	2.30	(0.74–7.11)	1.83	(0.55–6.06)
Other	1 / 10	10.0		0.70	(0.08–6.60)	0.88	(0.09–8.83)	0.84	(0.08–8.65)
White	6 / 44	13.6		1.00	(Reference)	1.00	(Reference)	1.00	(Reference)
HIV group			0.20						
Newly infected	15 / 93	16.1		1.30	(0.87–1.95)	1.23	(0.78–1.94)	1.30	(0.79–2.13)
Newly diagnosed	15 / 61	24.6		1.00	(Reference)	1.00	(Reference)	1.00	(Reference)
Antiretroviral therapy		0.76						
Yes	4 / 18	22.2		1.19	(0.36–3.90)	1.02	(0.27–3.89)	—	—
No	26 / 134	19.4		1.00	(Reference)	1.00	(Reference)	—	—
Gender of sex partners, past 12 months		0.45						
Men only	27 / 142	19.0		1.00	(Reference)	—	—	—	—
Men and Women	3 / 11	27.3		1.60	(0.40–6.42)	—	—	—	—
Transgendered sex partner, past 12 months		0.04						
Yes	6 / 15	40.0		3.17	(1.03–9.73)	3.37	(0.95–12.43)	5.15	(1.14–23.21)
No	24 / 138	17.4		1.00	(Reference)	1.00	(Reference)	1.00	(Reference)
Group sex			0.32						
Yes	13 / 54	24.1		1.51	(0.67–3.41)	—	—	—	—
No	17 / 98	17.4		1.00	(Reference)	—	—	—	—
Transactional sex, past 3 months		0.52						
Yes	4 / 16	25.0		1.41	(0.42–4.73)	—	—	—	—
No	26 / 136	19.1		1.00	(Reference)	—	—	—	—
Main partner, past 12 months		0.07						
Yes	8 / 64	12.5		0.44	(0.18–1.07)	0.34	(0.13–0.87)	0.39	(0.14–1.10)
No	22 / 90	24.4		1.00	(Reference)	1.00	(Reference)	1.00	(Reference)
Casual partner, past 12 months		0.02						
Yes	24 / 95	25.3		2.99	(1.14–7.82)	—	—	—	—
No	6 / 59	10.2		1.00	(Reference)	—	—	—	—
Anonymous partner, past 12 months		0.85						
Yes	7 / 38	18.4		0.91	(0.36–2.33)	—	—	—	—
No	23 / 116	19.8		1.00	(Reference)	—	—	—	—
**Substance use, past 3 months**								
Marijuana			0.36						
Yes	10 / 62	16.1		0.67	(0.29–1.16)	—	—	—	—
No	20 / 90	22.2		1.00	(Reference)	—	—	—	—
Cocaine			0.76						
Yes	4 / 23	17.4		0.83	(0.26–2.66)	—	—	—	—
No	26 / 129	20.2		1.00	(Reference)	—	—	—	—
Methamphetamine			0.34						
Yes	8 / 31	25.8		1.57	(0.62–3.96)	—	—	—	—
No	22 / 121	18.2		1.00	(Reference)	—	—	—	—
Inhalants			0.98						
Yes	1 / 5	20.0		1.02	(0.11–9.45)	—	—	—	—
No	29 / 147	19.7		1.00	(Reference)	—	—	—	—
Opiods			0.26						
Yes	2 / 5	40.0		2.83	(0.45–17.77)	—	—	—	—
No	28 / 147	19.1		1.00	(Reference)	—	—	—	—
Any drugs (excluding marijuana)		0.32						
Yes	13 / 54	24.1		1.51	(0.67–3.41)	2.25	(0.89–5.67)	2.44	(0.90–6.65)
No	17 / 98	17.4		1.00	(Reference)	1.00	(Reference)	1.00	(Reference)
Injection Drug Use, past 12 months		0.78						
Yes	2 / 12	16.7		1.25	(0.26–6.03)	—	—	—	—
No	28 / 140	20.0		1.00	(Reference)	—	—	—	—

Abbreviations. OR = Odds Ratio; CI = Confidence Interval; AOR = Adjusted Odds Ratio.

^Data represent mean and standard deviation.

Reporting a main partner decreased odds of drug resistance [adjusted odds ratio (AOR) 0.34; 95% confidence interval (CI) 0.13–0.87 with ARV users and marginally without the ARV users AOR 0.39, 95% CI 0.14–1.10], reporting a transgendered partnered increased odds (AOR = 3.37; 95% CI 0.95–12.43 with ARV users and AOR 5.15, 95% CI 1.14, 23.21 without ARV users); and being African American increased odds of drug resistance (AOR = 5.63, 95%CI 1.41–22.38 with ARV users and AOR 4.40, 95% CI 1.05, 18.40 without ARV users).

## Discussion

The prevalence of drug resistance of 19.5% among MSM in Los Angeles with new HIV diagnosis is relatively high compared to other reports [20] even when the analysis is restricted to those with acute/recent HIV infection (16.1%). This sample included those with new HIV diagnosis but no evidence of recent infection, therefore, some of this resistance may be acquired although there is evidence that transmitted resistance can persist for months or years in the absence of drug exposure[21, 22]. Concerns that this high level of resistance may be due to underreporting previous use of ARVs are lessened because of our study design: our subjects were recruited after seeking HIV testing on their own at a community clinic. This differentiates them from participants in other studies who were recruited from general community, not from among those who had sought testing on their own. General community samples captured individuals with HIV with a range of experience (for example in care, out of care but known positive, unknown positive)[23]. Because all participants in our study were obtaining HIV testing on their own initiative these individuals were subject to fewer motivations to underreport ARV use at the time of testing to affect their eligibility for a study enrollment (that included a financial incentive to participate). Moreover, all our participants completed their study questionnaires after they had been enrolled in the study, insuring their responses about ARV use did not affect their eligibility for the study. Reports of ARVs detected at relatively high levels in specimens of those reporting no ARV use and no knowledge of a previous diagnosis[8],[10] have come from intervention trials that incentivized HIV testing and determined study eligibility with further incentives based on a report of no ARV use and HIV results–a different source population and enrollment context from our study. Intervention trials that base receipt of monetary incentives on eligibility criteria are particularly vulnerable to inaccurate self-reports; the design of our study removed this potential reporting bias.

The increased risk of resistance among men who have sex with transgendered (TG) partners is a new finding. TGs represent a marginalized group of individuals among whom adherence to HIV treatment is particularly poor[24] and who may consequently be more likely to develop resistance. Because there was no difference by race/ethnicity for men reporting sex with TGs there does not appear to be an overlap in sexual networks of specific race/ethnicity groupings with those networks in which there are many TGs. There is also evidence HIV positive TG sex workers tend to use condoms less than MSM[25], suggesting another pathway that men who have sex with TGs may be more likely to acquire HIV. Our finding of this association of drug resistance with sexual exposure to TGs was strong; and remained the only factor associated with resistance except for race after we excluded those reporting use of ARVs from our analysis. TG represent a group who struggle with accessing and maintaining HIV care and services and this suggests their challenges may be contributing to specific transmission dynamics of resistant HIV.

Our findings point to the role of the main partner in providing protection against acquiring resistant virus and the increased risk with having casual partners. Clearly the more exposures, the more likely one of these is to someone with a resistant virus. Yet this also suggests that sexual networks are playing an increasingly important role in the transmission of resistance. It points to the need to counsel men without main partners that when they have unprotected sex with casual partners whose HIV status or use of therapy is unknown they may be at higher risk of acquiring resistant virus and that this has the potential to affect their therapeutic options.

Our finding that African American MSM were more likely than other MSM in Los Angeles to acquire resistance raises continuing concerns about the epidemic among African American MSM. These men are most likely to acquire HIV in general in the US–with African Americans representing half of all new infections in 2009[26] and African American MSM accounting for twice as many new infections as either white or Hispanic/Latino gay and bisexual men in 2010[27]. This suggests African American MSM may be having sexual encounters in very dense networks with high concordance of virus and also be in sexual networks of men with lower ARV adherence than other MSM, enhancing their likelihood of exposure to a resistant virus. A review of the literature of HIV among Black MSM in the US noted they were less likely to be adherent to HIV therapy than other MSM[28]. Finally, it should be noted that more of the African American MSM with drug resistance were in the newly diagnosed, not newly infected group, however, our analyses showed no significant difference by diagnosis timing.

Given the challenges of enrolling individuals into studies at the time of their HIV diagnosis and identifying those with acute or recent infection in any community, the sample size for this study was relatively small. This limited the power to more sensitively detect factors associated with resistance that were low prevalence. Although the study used computer-assisted interviewing and emphasized the conduct of the study in the most sensitive way for individuals with new HIV diagnosis, behavioral reports are always prone to mis-reporting and that could result in misclassification of participants in the analysis. Finally, the study and was initiated and tools developed in 2008 and earlier, when awareness of and well-developed tools for studying transgender individuals were not yet evolved fully limiting our ability to better study how them.

Our findings support the rationale for examining behavioral characteristics of resistance because we could not confirm findings from earlier studies that identified strong use, notably methamphetamine use, as a driver of multi-resistant HIV[29] suggesting a potential biological factor in drug use if it could be isolated from other behavioral factors. The role of substances on development of resistance requires further research but our findings suggest may not be a current priority. Phylogenetic studies have shown that certain strains of the virus can circulate in sexual networks identifiable by specific characteristics such as geography, age, substance use patterns, or other social characteristics. Resistant strains of the virus are likely doing the same; therefore understanding partner characteristics may point to networks with higher prevalence of such viruses and suggest prioritizing regular resistance testing on men from such networks. Finally, as individual characteristics may be of minimal use in identifying those more likely to have resistant virus, the characteristics of their partners may be a better marker of elevated risk and help focus counseling on the source of the resistance. Partner characteristics have been shown to be predictors of other risks such as acquisition of HIV and other STIs; our findings provide more evidence of the importance of studying such factors in the epidemiology of HIV and other STIs.

These findings indicate trends in HIV transmission patterns among MSM in Los Angeles. As a case of multi-drug resistant HIV has been documented among an individual on pre-exposure prophylaxis with emtricitabine (FTC)–tenofovir disoproxil fumarate (TDF) that was identified as transmitted and not acquired[30] there clearly remains a need to continue to monitor HIV resistance and factors associated with its transmission. A strength of our study is the ability to identify local factors associated with HIV acquisition and transmission of drug resistance in a community setting. Our sample was diverse by race/ethnicity and socio-economic status, and while all were MSM many reported female or transgender partners. Our findings indicate different transmission dynamics are emerging and the role of race/ethnicity and partnering patterns in the epidemic is becoming more apparent. African American MSM and TG individuals may have lower access to care and poorer adherence to therapy than other men, creating pockets of exceptional risk that will require special approaches to prevention and care to enhance their own health and reduce their likelihood to support transmission of drug resistance in the US.
